# Chemical bath deposition of textured and compact zinc oxide thin films on vinyl-terminated polystyrene brushes

**DOI:** 10.3762/bjnano.7.12

**Published:** 2016-01-25

**Authors:** Nina J Blumenstein, Caroline G Hofmeister, Peter Lindemann, Cheng Huang, Johannes Baier, Andreas Leineweber, Stefan Walheim, Christof Wöll, Thomas Schimmel, Joachim Bill

**Affiliations:** 1 Institute for Materials Science, University of Stuttgart, Heisenbergstrasse 3, D-70569 Stuttgart, Germany; 2 Institute of Functional Interfaces, Karlsruhe Institute of Technology (KIT), Hermann-von-Helmholtz-Platz 1, D-76344 Eggenstein-Leopoldshafen, Germany; 3 Institute of Applied Physics and Center for Functional Nanostructures, Karlsruhe Institute of Technology (KIT), Wolfgang-Gaede-Strasse 1, D-76131 Karlsruhe, Germany; 4 Institute of Nanotechnology, Karlsruhe Institute of Technology (KIT), Hermann-von-Helmholtz-Platz 1, D-76344 Eggenstein-Leopoldshafen, Germany; 5 Max Planck Institute for Intelligent Systems, Heisenbergstrasse 3, D-70569 Stuttgart, Germany

**Keywords:** bioinspired synthesis, polymer brush, template activation, thin film growth, zinc oxide

## Abstract

In this study we investigated the influence of an organic polystyrene brush on the deposition of ZnO thin films under moderate conditions. On a non-modified SiO*_x_* surface, island growth is observed, whereas the polymer brush induces homogeneous film growth. A chemical modification of the polystyrene brushes during the mineralization process occurs, which enables stronger interaction between the then polar template and polar ZnO crystallites in solution. This may lead to oriented attachment of the crystallites so that the observed (002) texture arises. Characterization of the templates and the resulting ZnO films were performed with ζ-potential and contact angle measurements as well as scanning electron microscopy (SEM), atomic force microscopy (AFM) and X-ray diffraction (XRD). Infrared spectroscopy (IR) measurements were used to investigate the polystyrene brushes before and after modification.

## Introduction

Due to its promising properties like photoemission in the UV range and its high piezoelectric coefficient, zinc oxide (ZnO; space group *P*6_3_*mc*) is interesting for a wide range of applications. Several research groups investigate its application in light emitting diodes, as surface acoustic wave generators or for field effect transistors [1–8]. Up to now, the fabrication of nanosized devices requires complex techniques like magnetron sputtering or pulsed laser deposition. Therefore, it is of high interest to develop easy-to-handle deposition processes for ZnO nanostructures. For example, biopolymers can control the mineralization and the structure formation of inorganic materials in an aqueous environment. Biopolymeric templates and their structure-inducing properties are in the focus of many recent works and issued in a recent collection [9–13]. With respect to ZnO, bio-templates are used for tailoring the morphology and crystallite sizes of ZnO [13–23], whereas n-type impurity dopants (e.g., Al^3+^, Ga^3+^ or In^3+^) have a significant influence on its optical properties [1–2,24–33]. In both cases, properties can be adjusted to match the requirements for different applications. Also the use of self-assembled monolayers (e.g., made from 1-thioacetato-16-(trichlorosilyl)hexadecane [34–35] or 3-aminopropyltriethoxysilane [33]) and polyelectrolytes [36] as organic templates are known, which can modify the surface charge on the substrates for ZnO precipitation under moderate conditions.

Based on these studies, we investigated the ZnO film formation on silicon wafers modified with vinyl-terminated polystyrene (PS) brushes. Those brushes consist of PS molecules that are grafted to a silicon wafer forming a very thin film. The obtained ZnO thin films, which were synthesized via chemical bath deposition (CBD) at 60 °C, were characterized by X-ray diffraction (XRD), scanning electron microscopy (SEM), AFM and infrared spectroscopy (IR). Our results imply an activated dynamic precipitation model of ZnO thin films, which is the result of a base-catalyzed transesterification of polystyrene brushes in the early stage of ZnO precipitation.

## Results and Discussion

### Template characterization

**Scheme 1:**
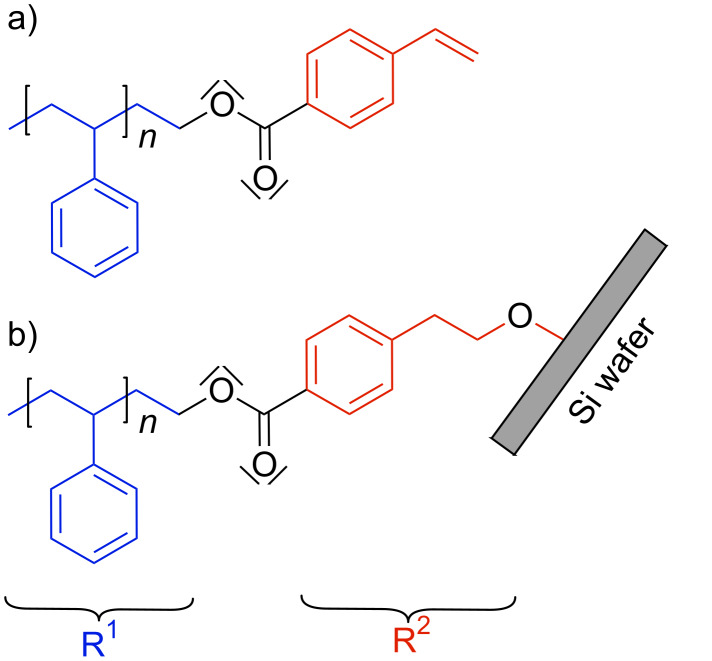
a) Structural formula of the α-methyl-ω-*p*-vinyl-benzoate-polystyrene molecule. b) Schematic representation of a PS molecule after brush formation via a condensation reaction.

For the deposition of the PS brush, the molecule depicted in Scheme 1a was grafted to a cleaned Si wafer. Maas et al. [37] found that the functional vinyl endgroup is transformed into an alcohol which then can react in a condensation reaction with silanol groups at the SiO*_x_* surface (Scheme 1b).

**Figure 1:**
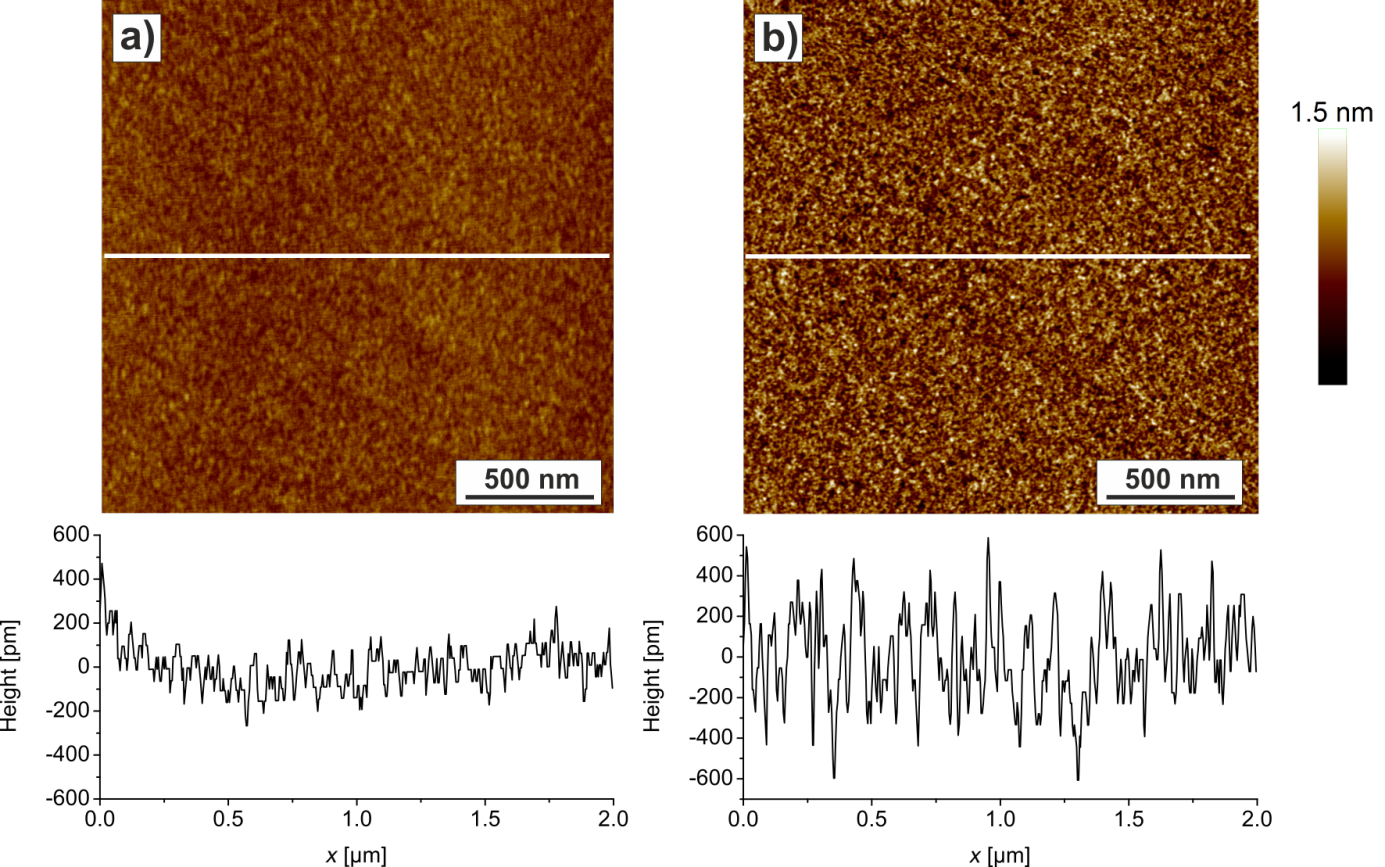
AFM height images and corresponding cross sections of a) a Si wafer and b) a PS brush.

Prior to the ZnO deposition experiments, Si wafers before and after PS brush formation were characterized. Figure 1 shows AFM images and cross sections of the two different substrates. The SiO*_x_* as well as the PS brush have a low roughness of 0.2 nm. After coating the highly polished SiO*_x_* surface with the PS brush, a height profile with more pronounced peaks is obtained. Since the conformation of the polymer chains is governed by the surroundings, a more irregular structure arises. The wafer is homogeneously covered with the polymer template and no holes or defects are visible (Figure 1b). A thickness of 1–2 nm of the PS brush was measured by scratching the template with an AFM tip. This data are consistent with ellipsometry measurements (not shown) performed with a single-wavelength ellipsometer (DRE-ELX-02, DRE, Germany). Taking into account the molecular weight of the polymer (*M*_w_ = 2600 g·mol^−1^) and the monomers weight of 104.15 g·mol^−1^, the molecules consist of about 25 monomers. Assuming a monomer length of 0.2 nm, the length of a fully stretched polymer chain would be about 5 nm. Together with the specific weight of polystyrene (1.05 g·cm^−3^), a density of 0.822 molecules per nm^2^ can be calculated for this densely packed perpendicular arrangement of the molecules. Since we measured a thickness of 1–2 nm for our PS films, we can calculate a nominal grafting density of 20–40% (0.164–0.329 molecules per nm^2^) for our brush system.

**Figure 2:**
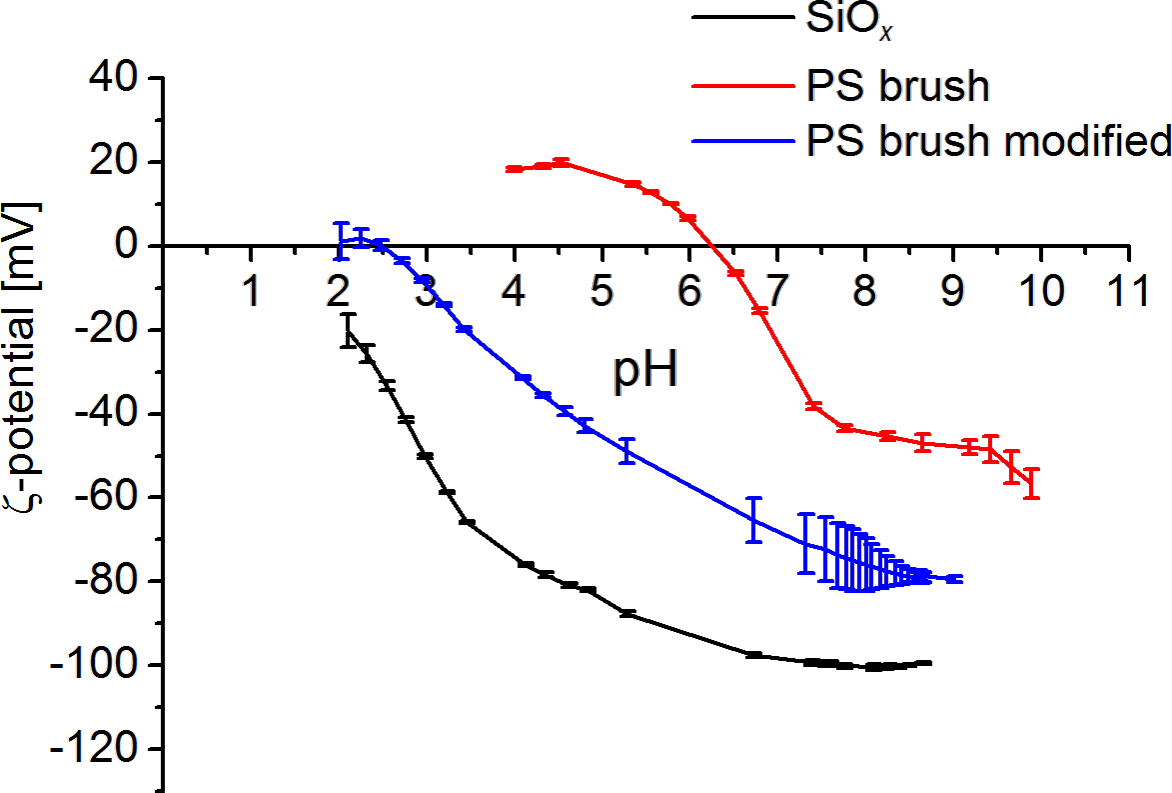
ζ-potential of the Si wafer and the PS brush before and after modification measured in water.

The polarity of the different surfaces was investigated by ζ-potential and contact angle measurements. The isoelectric point (IEP), measured in water, of the plasma-cleaned SiO*_x_* surface and the PS brushes is 1.5 (extrapolated) and 6.3, respectively (Figure 2). At pH 9 of the reaction solution, the ζ-potential of the Si wafer is much lower (ca. −100 mV) than that of the brushes (ca. −50 mV). This means that the surface charge of the SiO*_x_* is highly negative whereas under the same conditions, the PS brush is much less negatively charged. It is noteworthy that the reaction takes place in methanol instead of water. For mixtures of water and alcohol it is known, that the ζ-potential is decreasing with higher alcohol content [38]. This could lead to a smaller difference in the ζ-potential of the SiO*_x_* and PS brush in methanol as compared to the results in water.

Water contact angle (WCA) measurements confirm the findings of the ζ-potential measurements. The SiO*_x_* surface exhibits a high hydrophilicity with a WCA below 4°. On the other hand, the surface decorated with a PS brush is hydrophobic with a WCA of 85°.

### Transesterification of polystyrene brushes

**Scheme 2:**
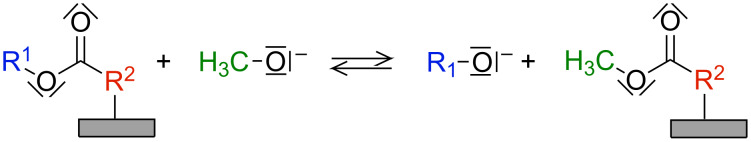
Mechanism of the proposed transesterification process, which modifies the polystyrene brush in the methanolic ZnO deposition solution with an alkaline pH at elevated temperatures.

During the ZnO mineralization, a modification of the PS brush was observed that leads to a hydrophilic surface, which can improve the interaction with the ZnO nanoparticles in solution. In the methanolic medium with an alkaline pH at elevated temperatures, a transesterification can take place as indicated in Scheme 2. This would result in a separation of the polystyrene chain (R^1^ in Scheme 1 and Scheme 2) from the short end group (R^2^ in Scheme 1 and Scheme 2) attached to the SiO*_x_* surface. A polar ester group, which was previously shielded by the non-polar polymer chain, now terminates the remaining molecule. To obtain further information about this reaction, several experiments were carried out. The PS brushes as well as the polymer powder were treated in a modified reaction solution without zinc acetate to prevent ZnO deposition.

ζ-potential and water contact angle measurements on the modified brushes confirm the formation of a hydrophilic surface after the treatment. The ζ-potential decreases from −50 mV to −80 mV as shown in Figure 2. The contact angle decreases to a value of 60°. On the AFM images (not shown), no change is visible before and after modification.

**Figure 3:**
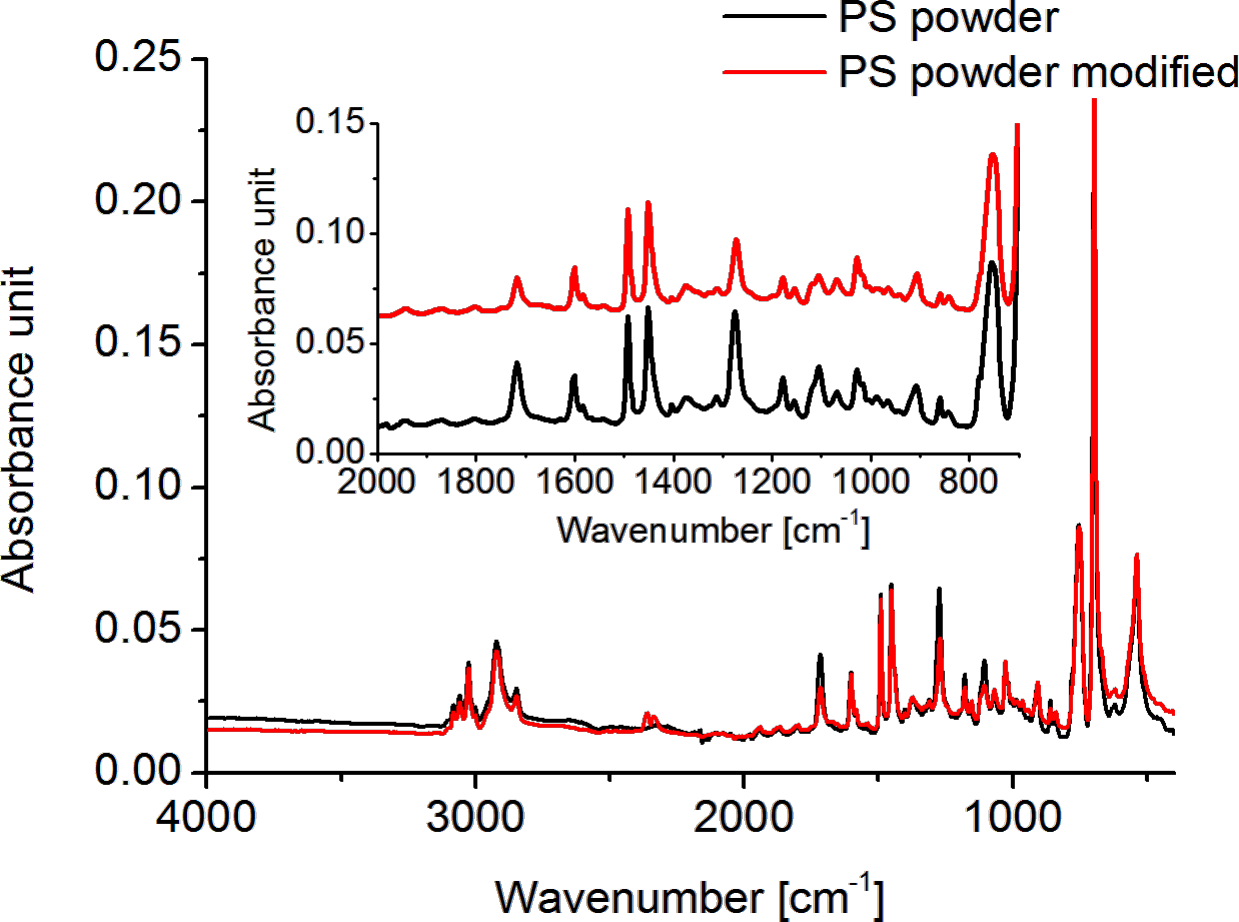
ATR spectra of PS powder used for the preparation of the brushes before and after modification. The inlay shows a magnification of the range between 700 and 2000 cm^−1^. After the modification, the bands attributed to COC vibrations decrease in intensity.

**PS powder:** Attenuated total reflection (ATR) measurements were performed on the α-methyl-ω-*p*-vinyl-benzoate-polystyrene powder used to prepare the brushes. Figure 3 shows the spectra obtained before and after treatment in the modified reaction solution. Characteristic bands originating from PS [39] can be found for both samples and are listed in Table 1. Some additional bands can be assigned to the ester group present in the polymer chain (Table 2).

**Table 1:** Bands of PS found in the ATR spectra and their assignment.

wavenumber [cm^−1^]	assignment [39]

3028–3026	aromatic H-stretching modes
2922–2849	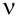 (CH_2_)
1600–1311	aromatic vibrations
1070–1028	aromatic H-bending modes
908	aromatic vibrations
754	aromatic vibrations

**Table 2:** Characteristic IR bands of the ester group.

wavenumber [cm^−1^]	assignment [40]

1716	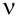 (CO)
1274	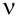 _as_(COC)
1107	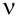 _s_(COC)
860	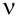 (COC)

During the transesterification, the PS molecule is split into two parts. The smaller part R^2^ with the ester group is soluble in methanol, whereas the longer chain R^1^ remains insoluble. Due to the washing steps during modification, the smaller molecules are removed from the reaction vessel. Therefore, the number of ester groups present in the powder should decrease after the treatment. Indeed, the intensities of the corresponding bands in the ATR spectra decrease as well. The intensity of the bands coming from the phenyl groups on the other hand nearly remains the same. The amount of phenyl groups in the end groups is very small compared to those in the polymer chain (ratio about 1:25). The decrease in intensity is therefore very small. Additionally, the transesterification seems to be incomplete, since the ν(COC) bands do not disappear completely after three modification cycles.

**PS brush:** In order to prove our conclusions, infrared reflection absorption spectroscopy (IRRAS) measurements were performed on a PS brush grafted to the Si wafer. In this case, the main part of the chain detaches from the surface and only the small rest with the ester group remains, since it is covalently bound to the substrate (compare Scheme 1b). Indeed, the spectra (see Supporting Information File 1) show a decrease in intensity for the aromatic bands after the modification reaction, confirming the ATR results. Here it also seems that the transesterification process is incomplete and a PS Brush with modified properties (increased polarity and charge) is the result.

### XRD investigation

**Figure 4:**
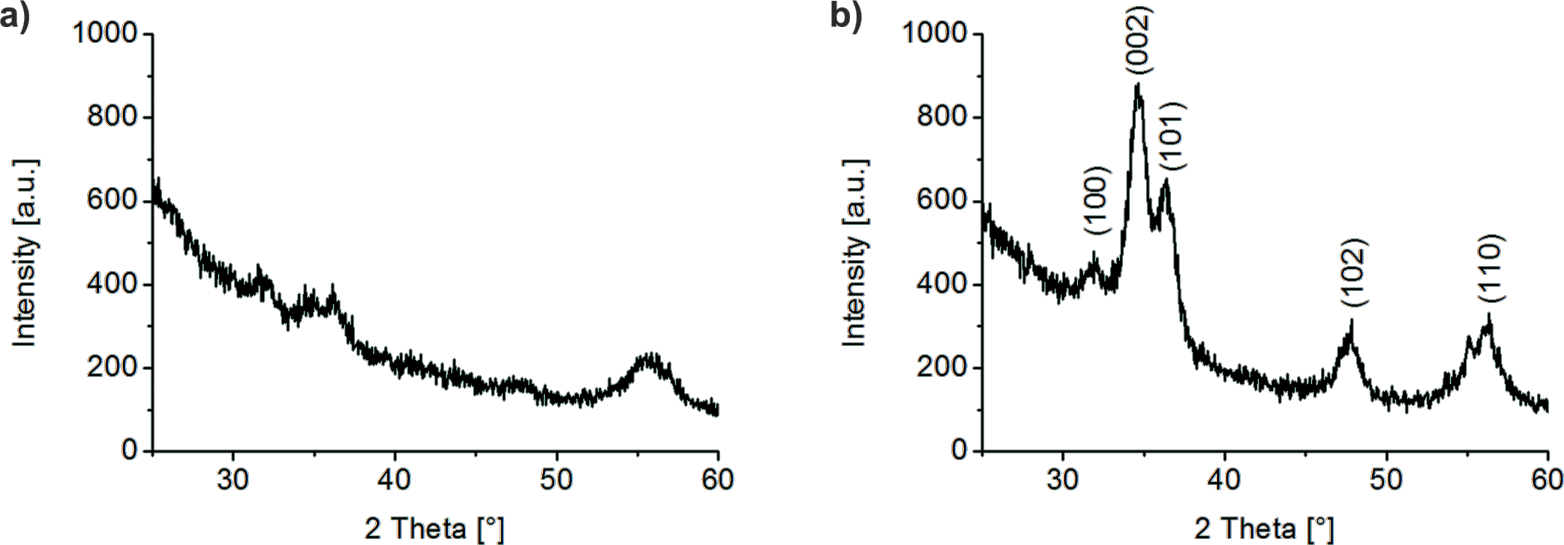
XRD diagrams of ZnO films deposited on a) SiO*_x_* and b) PS brushes after 20 mineralization cycles. The ZnO film on the PS brushes shows a preferred crystal orientation along the (002) direction.

ZnO films were deposited within methanolic solution under moderate condition (60 °C) on SiO*_x_* and as-prepared PS brush. The obtained ZnO thin films were characterized by XRD measurements of the 20 mineralization cycles sample (Figure 4). As expected, the characteristic (100), (002), (101), (102) and (110) reflections of hexagonal ZnO are visible in the XRD patterns (cf. JCPDS no. 01-079-0206). For the PS brush sample (Figure 4b), a pronounced preferred orientation of the ZnO crystallites with the hexagonal c-axis perpendicular to the plane of the Si substrate is indicated by the strong (002) reflection within the XRD pattern. The corresponding film grown on SiO*_x_* on the other hand shows no texture (Figure 4a). A value for the average crystallite size of precipitated ZnO on PS brushes was determined to be around 4.2 ± 0.1 nm. This calculated crystallite size is in the range of similar systems prepared by similar procedures [33–35].

### AFM and SEM results

**Figure 5:**
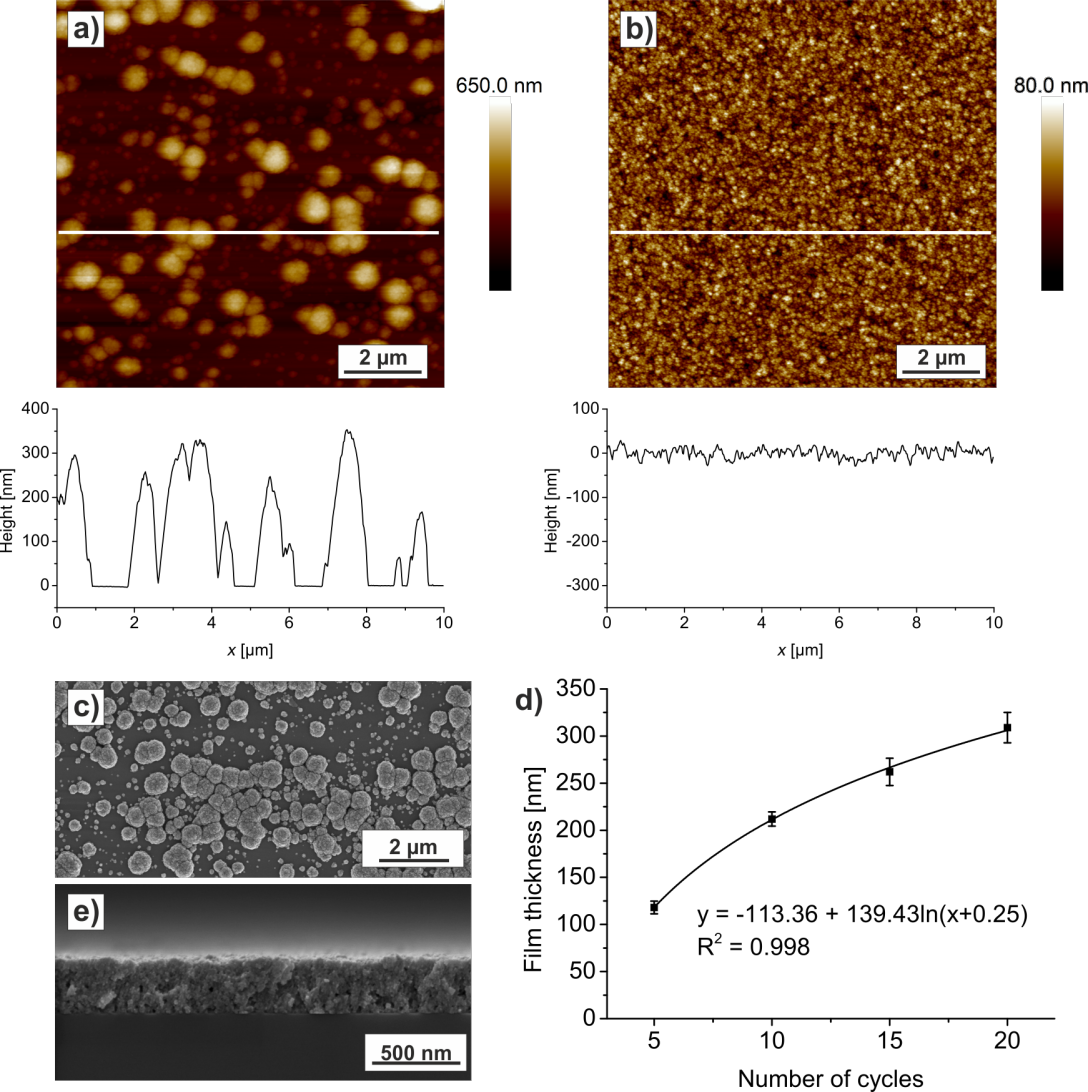
a) AFM topography and cross section of ZnO islands deposited on SiO*_x_* after 20 mineralization cycles. The corresponding SEM top view is shown in c). b) AFM topography and cross section of ZnO film deposited on PS brush after 20 mineralization cycles. d) Film thickness of the films on PS brush measured from SEM cross sections plotted against the number of mineralization cycles. e) SEM cross section of the film on PS after 20 mineralization cycles.

The characterization of the surface and structure was done with AFM and SEM measurements. In the case of SiO*_x_* as substrate, big islands of ZnO can be seen on the AFM- (Figure 5a) and SEM- (Figure 5c) images of the 20 cycle sample. This Volmer–Weber-like growth [41] indicates that the surface energy of Si is smaller than the interface energy between ZnO and SiO*_x_* plus the surface energy of ZnO.

In the case of the PS template, the growth of a homogeneous film is favored (Figure 5). A representative cross section of a ZnO film grown on PS is shown in Figure 5e. In Figure 5d the film thickness is plotted in dependence on the number of deposition cycles. The growth rate seems to decrease with increasing film thickness. This might indicate a stronger interaction of the particles in solution with the template compared to the interaction with the already deposited ZnO film. This strong interaction with the substrate also supports our conclusion that the PS brush is modified in the deposition solution towards a functionalized surface.

### Mineralization mechanism

By correlating the transesterification of the PS brush with the observed homogeneous ZnO mineralization, the deposition process can be explained. We put forward the following growth model: At the beginning of the mineralization process, some few PS molecules are modified by the transesterification. As it was shown by Turgeman et al. [42–43], the polar ZnO particles can interact with the ester groups during the mineralization. Therefore, they act as preferred sites for deposition of ZnO particles. With increasing reaction time, the number of available reaction sites is increasing as more and more PS molecules are removed. After a few deposition cycles, a complete ZnO film is formed. The methyl ester can coordinate the polar ZnO crystallites. This in turn can support an oriented attachment of the nanoparticles to the surface and an anisotropic orientation of the entire film, which was observed by XRD (Figure 4) [42–43].

## Conclusion

ZnO thin films were grown by chemical bath deposition under moderate conditions. On a SiO*_x_* surface, island growth of the ZnO was observed. A polystyrene brush on the other hand acts as a structure-inducing template and leads to the formation of a homogeneous, compact film that shows a preferred crystal orientation along the (002) direction. IR spectroscopy measurements indicate that the nonpolar PS brush partially decomposes via a transesterification reaction, resulting in a polar surface. This new surface interacts with the ZnO crystallites, which are simultaneously formed within the solution and assemble in an anisotropic fashion, forming a compact ZnO film.

## Experimental

### Preparation of the PS brush

Silicon wafers with a surface orientation of [100] and a native oxide layer with 2–3 nm thickness (Wacker Burghausen, Germany) were used as substrates. Before cleaning them with a CO_2_ snow jet [44] in order to remove organic residues, they were cut into pieces of 10 mm × 20 mm. Now, approximately 100 µL of a solution (3% by weight in toluene) of α-methyl-ω-*p*-vinylbenzoate-polystyrene molecules (vinyl-terminated PS, Polymer Standard Source, Canada, *M*_w_ = 2600 g·mol^−1^; *M*_n_ = 2400 g·mol^−1^) was cast on the surfaces and left evaporating. This process was repeated three times, yielding a final film thickness of several micrometers. The samples were then placed into a massive aluminum vacuum chamber, pumped with a scroll vacuum pump with a pressure of less than 1 mbar. The chamber was placed onto a hot plate at 145 °C for 12 h [37]. Excess molecules were removed by a 3-step counterflow rinsing (cascaded rinsing) of the samples in tetrahydrofuran (THF). The samples were dried by a stream of nitrogen after each rinsing process.

### Preparation of the deposition solution and mineralization

Deposition experiments of ZnO films were carried out as described by Eisele et al. [33]. Stock solutions of polyvinylpyrrolidone (*M*_w_ = 10,000 g·mol^−1^, Lot#BCBF4168V, Sigma-Aldrich), zinc acetate (ZnAc_2_, Zn(CH_3_COO)_2_·2H_2_O, puriss p.a., ACS reagent, ≥99.0%, Sigma-Aldrich) and tetraethylammonium hydroxide (TEAOH, 1.5 M in methanol, Sigma-Aldrich) in methanol (VLSI Grade, J. T. Baker) with concentrations of 20, 40 and 85 mM, respectively, were mixed. The reaction solution was prepared by mixing the stock solutions in a volume ratio of PVP/ZnAc_2_/TEAOH 3:2:2. Thereby, the TEAOH solution was added drop wise to the PVP–ZnAc_2_ mixture with a peristaltic pump at a flow rate of 1.044 mL·min^−1^ under gentle stirring. The reaction solution was prepared anew every day to prevent agglomeration of particles.

The functionalized Si wafers and the reference samples without brush were immersed in 1 mL of the precursor solution in a closed vessel, each. The vessels were heated within an oil bath at 60 °C. After 1.5 h, the substrates were washed abundantly in methanol and dried with N_2_. For each cycle, a new vessel and fresh solution was used. To get thicker films, several cycles were carried out.

For the investigation of the transesterification process of the brushes during mineralization, a modified deposition solution was used. To prevent ZnO formation, the ZnAc_2_ stock solution was replaced by pure methanol. The modification was performed according to the standard deposition experiments for three cycles.

### Powder X-ray diffractometry

Grazing incidence X-ray diffraction measurements with an incidence angle of 2° were performed using an X’Pert MRD (PANalytical) using Cu Kα radiation parallelized with polycapillary optics and using a flat graphite monochromator in the diffracted beam in a diffraction angle, 2θ, range of 25 to 60°. Data evaluation was done using a full-pattern Pawley refinement on the basis of the diffraction data using the TOPAS software [45]. For that the instrumental profile as determined from a LaB_6_ powder standard (Standard Reference Material SRM 660a, National Institute of Standard and Technology NIST, Gaithersburg, USA) was fitted and taken fixed for evaluation of the diffraction data from the ZnO. The line broadening of the latter was evaluated by refining Lorentzian-shaped size broadening having, for each hkl, an integral breadth on the diffraction angle scale, β_2θ_, of

[1]
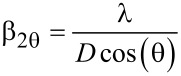


Equation 1 basically corresponds to Scherrer’s equation with λ as the wavelength of radiation and with *D* as an average of the crystallite size.

### Scanning electron microscopy

Micrographs were taken with a DSM 982 Gemini (Zeiss). An accelerating voltage of 3 kV and a working distance of 2–3 mm were used. To ensure conductivity, the samples were sputtered with a 0.8 nm thick layer of Pt/Pd (80:20). The thicknesses of ZnO films were determined using freshly prepared cross sections.

### Atomic force microscopy

AFM measurements were performed on a Bruker Multimode 3 using commercially available cantilevers (PPP-NCHR, Nanosensors).

### Water contact angle measurements

The static contact angle of Milli-Q water on the modified and unmodified samples were measured on a Contact Angle Measurement System G10 from Kruess. The results are an average of at least five measurements.

### Zeta-potential measurements

Measurements of the ζ-potential were performed on a SurPASS Electrokinetic Analyzer (Anton Paar GmbH). Samples were glued on the stamps of an adjustable gap cell (10 mm × 20 mm) with double-sided tape and a gap height of 100 µm was used. A 1 mM KCl solution was purged with nitrogen prior and during the measurements. For automatic titration, a 0.1 M HCl solution was used. For each measurement point, four pressure ramps from 0 to 400 mbar were performed and the streaming current was measured. The ζ-potential was calculated with a Fairbrother–Mastin approach.

### Infrared spectroscopy

**IRRAS:** The IRRA-spectra were recorded on a Bruker Vertex 80 purged with dry air under a fixed angle of incidence of 80°. The data were collected on a narrow band liquid nitrogen cooled mercury cadmium telluride (LN-NB-MCT) detector with a resolution of 2 cm^−1^. Perdeuterated hexadecanethiol-SAMs on Au/Ti/Si wafer were used for reference measurements and 1024 scans were taken. To enhance the signal-to-noise ratio, PS brushes grafted to a Si-wafer coated with 10 nm SiO_2_, 100 nm Au and 5 nm Ti as adhesive layer between Au and Si were prepared [46]. For the sample measurement between 900 and 1300 scans have been cumulated, the spectra were recorded until no water bands could be observed in the spectra. The absorption band positions are given in wavenumbers 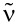 in cm^−1^.

**ATR:** PS powder used to prepare the brushes was treated in the modified reaction solution. After each cycle, the powder was washed with methanol. Untreated powder was used for reference measurements. The ATR spectra were recorded on a Bruker Tensor 27 with Platinum ATR accessory. The data were collect on a room temperature deuterated L-alanine doped triglycine sulfate (RT-DLaTGS) detector with a resolution of 4 cm^−1^. The empty diamond crystal was measured against air as background. For background and sample 64 scans have been recorded.

## Supporting Information

File 1 Results of IRRAS measurements on the PS brushes grafted to Si before and after modification.
