# Supplementary material for: Chemical bath deposition of textured and compact zinc oxide thin films on vinyl-terminated polystyrene brushes
Source: Beilstein J Nanotechnol. 2016 Jan 25;7:102–10. doi: 10.3762/bjnano.7.12 (PMC4734420; doi:10.3762/bjnano.7.12)
Supplement: File 1 — Results of IRRAS measurements on the PS brushes grafted to Si before and after modification. [file Beilstein_J_Nanotechnol-07-102-s001.pdf]

Supporting Information

for

Chemical bath deposition of textured and compact zinc oxide thin films on vinyl-terminated polystyrene brushes

Nina J. Blumenstein,[1] Caroline G. Hofmeister,[1] Peter Lindemann,[2] Cheng Huang,[3,4] Johannes Baier,[1] Andreas Leineweber,[5] Stefan Walheim*[3,4] Christof Wöll,[2] Thomas Schimmel[3,4] and Joachim Bill*[1]

Address: [1]Institute for Materials Science, University of Stuttgart, Heisenbergstrasse 3, D-70569 Stuttgart, Germany and [2]Institute of Functional Interfaces, Karlsruhe Institute of Technology (KIT), Hermann-von-Helmholtz-Platz 1, D-76344 Eggenstein-Leopoldshafen, Germany and [3]Institute of Applied Physics and Center for Functional Nanostructures, Karlsruhe Institute of Technology (KIT), Wolfgang-Gaede-Strasse 1, D-76131 Karlsruhe, Germany and [4]Institute of Nanotechnology, Karlsruhe Institute of Technology (KIT), Hermann-von-Helmholtz-Platz 1, D-76344 Eggenstein-Leopoldshafen, Germany and [5]Max Planck Institute for Intelligent Systems, Heisenbergstrasse 3, D-70569 Stuttgart, Germany

Email: Joachim Bill* - bill@imw.uni-stuttgart.de;
Stefan Walheim* - stefan.walheim@kit.edu

*Corresponding author

Results of IRRAS measurements on the PS brushes grafted to Si before and after modification

In order to obtain more information about the transesterification, infrared reflection absorption spectroscopy (IRRAS) measurements were performed on polystyrene brushes (Figure S1). The bands at 2928 and 2850 cm^{-1} can be assigned to the CH_2-asymmetric and symmetric stretching modes $\nu(CH_2)$ of the backbone, respectively. A band arising from aromatic H-stretching mode can be found at 3025 cm^{-1}. A large band at 1688 cm^{-1} originating from amide I indicates that there seems to be some residual polyvinylpyrrolidone present. Comparing the relative intensities of the bands at 3025 and 2928 cm^{-1} before and after treatment indicates a decrease in relative intensity for the aromatic band. This is expected, since the main part of the molecule is being removed from the substrate surface during the modification.

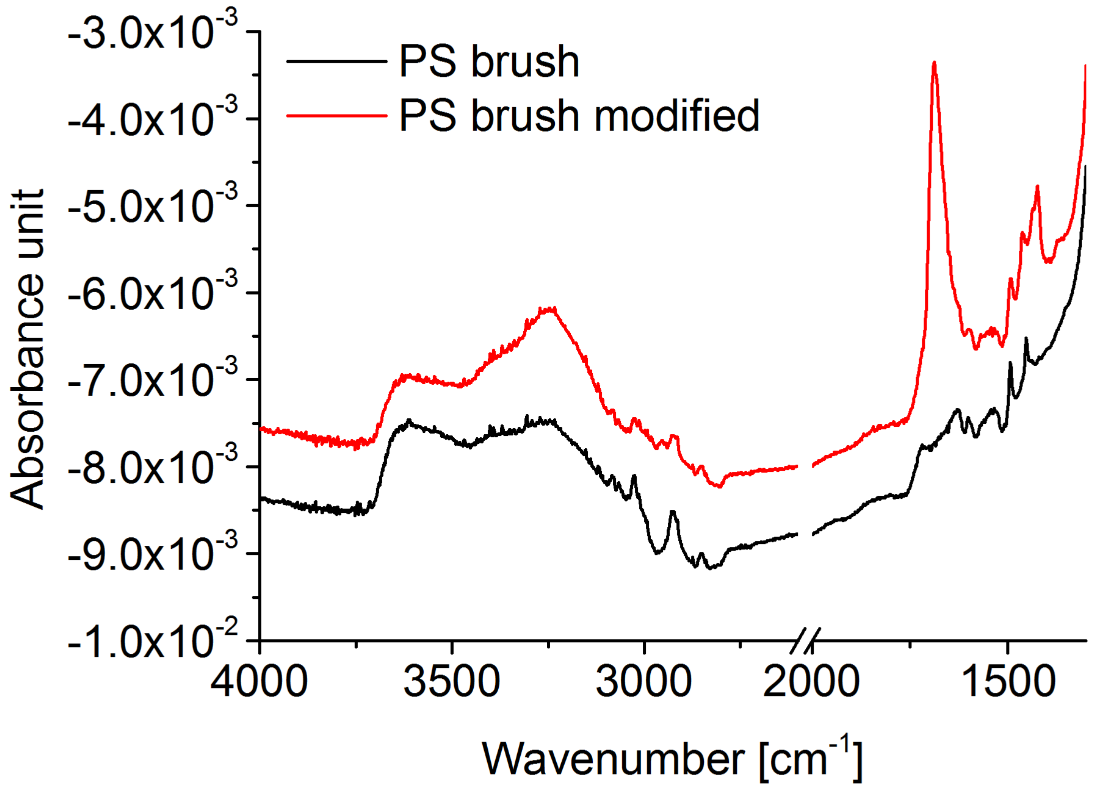

Figure S1: IRRAS spectra of PS brushes before and after modification.
